# Long noncoding RNA DANCR regulates *miR-1305*-Smad 4 axis to promote chondrogenic differentiation of human synovium-derived mesenchymal stem cells

**DOI:** 10.1042/BSR20170347

**Published:** 2017-07-21

**Authors:** Lei Zhang, Xiangyi Sun, Shuo Chen, Chao Yang, Ben Shi, Liwu Zhou, Jianning Zhao

**Affiliations:** 1 Department of Orthopedics, Jinling Hospital, Nanjing University, School of Medicine, Nanjing, China; 2 Department of Orthopedics, Nanjing Jiangbei People’s Hospital, Nanjing, China; 3 Department of Orthopedics, Nanjing General Hospital, The Second Military Medical University Clinical Medical School of Nanjing, Nanjing, China

**Keywords:** chondrogenesis, DANCR, miR-1305, Smad4, SMSC

## Abstract

miRNAs have been reported to regulate cellular differentiation by modulating multiple signaling pathways. Long noncoding RNA (lnc RNA) DANCR was previously identified to be critical for the chondrogenesis of human synovium-derived mesenchymal stem cells (SMSC), however, the underlying molecular mechanism requires better understanding. Here, miRNA expression profiling in DANCR overexpressed in SMSCs identified significant down-regulation of *miR-1305*, which serves as a downstream target of DANCR. Notably, *miR-1305* overexpression reversed DANCR-induced cell proliferation and chondrogenic differentiation of SMSCs, which suggested that *miR-1305* antagonized the function of DANCR. Mechanistically, highly expressed *miR-1305* resulted in the decreased expression of the TGF-β pathway member Smad4, and inhibition of *miR-1305* enhanced the expression level of Smad4. Depletion of Smad4 suppressed the promotion of DANCR in cell proliferation and chondrogenesis of SMSCs. Collectively, our results characterized *miR-1305*-Smad4 axis as a major downstream functional mechanism of lncRNA DANCR in promoting the chondrogenesis in SMSCs.

## Introduction

Osteoarthritis is a chronic, progressive, and degenerative form of arthritis, which remains a major clinical challenge due to the limited self-repair capacity of cartilage. Developing alternative treatment strategy in the repair of damaged cartilage is quite necessary. Autologous chondrocyte implantation has been applied widely with confirmed clinical effects in terms of repairing cartilage defects [1–4]. However, due to the limited donor sources of autologous chondrocytes and the dedifferentiation of *in vitro* cultured chondrocytes, the clinical application of autologous chondrocyte repair is limited [5].

Mesenchymal stem cells (MSCs), which are somatic cells with an unlimited capacity for self-renewal and differentiation under appropriate conditions, have become a strong candidate for tissue engineering to regenerate cartilage due to their ease of isolation and amenability to *ex vivo* expansion [6–8]. It is documented that MSCs directly act in cartilage formation, as well as release trophic factors, and promote angiogenesis [9–11]. Amongst the various available sources, MSCs seem to have many advantages over their counterparts, while recent study demonstrated that synovium-derived MSCs (SMSCs), which have better chondrogenic potential compared with MSC, are gaining momentum [12–17].Improved MRI features, histology, and better clinical outcome have been achieved in cartilage repair derived from SMSCs [14]. These findings shed light on the potential application of SMSC in the field of chondrogenesis, and understanding the molecular mechanism in cartilage repair will benefit the use of SMSCs.

Long noncoding RNAs (lncRNAs) are defined as RNA species >200 nts with no protein-coding function, which play important roles in mediating cell proliferation and differentiation [18,19]. Dysfunction of lncRNA has been observed in a variety of human diseases [19–21]. Our previous data demonstrated that lncRNA DANCR, which was first identified in hepatocellular carcinoma (HCC) [22], promoted the cell proliferation and chondrogenic differentiation through up-regulating the expression of Smad3 and STAT3 [12]. These results provided one of the mechanisms for the role of SMSC in cartilage repair. Additionally, increasing evidence indicated the essential role of miRNAs in modulating cellular differentiation, which are key regulators in tissue development and homeostasis [23–28]. These miRNAs are typically 20–22 nts in length generated via a stem-loop structure by the Dicer complex [25]. Mature miRNAs regulate genes through complementary interactions with the 3′-UTR region of mRNA, resulting in the degradation of mRNA or inhibition of protein translation [25]. Several miRNAs have been demonstrated to be involved in chondrogenic differentiation of MSCs [13,29–33]. Amongst these, *miR-410* was reported to promote the chondrogenic differentiation through Wnt signaling pathway [34]. *MiR-495* inhibits chondrogenesis in human MSCs by targetting SRY-box 9 (Sox9) [30]. These findings indicated the important roles of miRNAs in chondrogenesis.

In the present study, we performed miRNA expression profiling to illustrate the candidate miRNAs that were regulated by lncRNA DANCR. Our result demonstrated that highly expressed DANCR results in the decreased expression of *miR-1305*, which consequently negatively regulated the expression of Smad4. Our results characterized the negative-regulation loop between DANCR and *miR-1305*. These data provide the mechanism of DANCR in chondrogenic differentiation.

## Materials and methods

### SMSCs isolation

The assay was approved by the local Clinical Research Ethics Committee and performed in accordance with The Code of Ethics of the World Medical Association (Declaration of Helsinki). Informed consents were obtained from the patients. The protocol for SMSCs isolation was established as previously described [13]. Briefly, synovial tissue was collected from the patients with osteoarthritis during the operations. The tissues were cut into small pieces, washed with PBS, and cultured in DMEM medium containing 10% FBS (Invitrogen, U.S.A.), 1% penicillin/streptomycin (Invitrogen), 3% collagense P (Roche, Mannheim, Germany), and 0.5% gentamycin (Biochrom). Tissues were digested at 37°C for 3 h, and then the suppression was centrifuged and seeded in expansion medium DMEM with 10% FBS (Gibco, U.S.A.).

### Construction of DANCR expression vector

The cDNA sequence of DANCR was amplified by PCR with the primers (forward: 5′-CTCGGAGGTGGATTCTGTTAG; reverse: 5′-CTGCAGAGTATTCAGGGTAAGG). The PCR products were digested with the enzymes BgIII and XhoI. The sequence was inserted into the pcDNA3.1 expression vector by T4 DNA ligase. The insertion was verified by sequencing.

### Nude mice tumorigenesis

SMSCs (2 × 10^7^) stably expressing DANCR was subcutaneously injected into the left flanks of the nude mice (NOD/SCID, 5 weeks of age, female, 18.6 ± 0.2 g). The tumorigenicity was monitored once per week. The mice were killed 3 months after the injection. This experiment was approved by the Laboratory Animal Center of Nanjing University and complies with the National Institutes of Health Guide for the Care and Use of Laboratory Animals (NIH publication number 8023, revised 1978).

### Proliferation assay

The cell counting kit-8 (CCK-8; Dojindo Molecular Technologies, Inc., Rockville, MD, U.S.A.) was used to detect the cell number. SMSCs were seeded in the 96-well plate at a density of 2 × 10^3^ per well. When the cell confluence reached 80–90%, 10 μl CCK-8 reagent was added to the culture medium and incubated at 37°C for 3 h. The absorbance of each well at 450 nm was determined with the Absorbance Microplate Reader (Bio–Rad).

**Cell cycle and cell division assay**

SMSCs transfected with control vector or DANCR were harvested and subjected to trypsinization. Cells were washed with PBS and fixed in 75% ethanol at 4°C overnight. Afterward, cells were incubated with 1 mg/ml RNase A at 37°C for 30 min, and then the cells were stained with 50 µg/ml propidium iodide (PI). The cell cycle distribution was analyzed by flow cytometry. Cell division assay was performed by flow cytometry with carboxyfluorescein diacetate succinimidyl ester (CFSE) cell division kit (10009853, Cayman Chem) according to the manufacturer’s instructions. Briefly, SMSCs were washed with prewarmed PBS and centrifuged at 300 ***g*** for 5 min. The cell pellets were resuspended at a density of 1–2 × 10^7^ cells/ml with PBS. An equal volume of 2× CFSE staining solution was added into the cell suspension and incubated at 37°C for 15 min. Afterward, an equal volume of DMEM medium containing 10% FBS was added and centrifuged the cells at 300 ***g*** for 5 min at room temperature. The cells were washed with 15-ml culture medium three times. Cells were resuspended and cultured for the indicated time. To detect the dilution of CFSE, cells were harvested and the signal was monitored by flow cytometer with excitation at 488 nm and emission at 525 nm.

### *In vitro* chondrogenic differentiation assay

SMSCs stably expressing DANCR or control vector were cultured in 15-ml polypropylene tubes. Cells were harvested and centrifuged at 500 ***g*** for 15 min. The pellets were cultured in high-glucose DMEM, which contained 100 nM dexamethasone, 50 μg/ml ascorbate-2-phosphate and 50 mg/ml ITS + TMP remix (Becton Dickinson) for 14 days.

### Real-time quantitative RT-PCR analysis of miRNA

miRNA was extracted using the miRcute miRNA Isolation Kit (DP501, TIANGEN Biotech (Beijing) Co., Ltd.). The first cDNA strand was synthesized using the miRcute miRNA cDNA Synthesis Kit (KR201, TIANGEN Biotech (Beijing) Co., Ltd.) according to the manufacturer’s instructions. Quantitative real time-PCR was conducted with the ABI Stepone Plus Real-time PCR platform using SYBR Green I PCR reagents (Toyobo, Osaka, Japan). U6 was used as the normalization control. The relative expression level of *miR-130a, miR-145*, and *miR-1305* were calculated with the 2^–ΔΔ*C*^_t_ method. Primer sequences were listed as follows: *miR-130a*, forward: 5′- GTCGTATCCAGTGCGCUCUUUUACAUUCUCUAATTGCACTGGATACGACCGCATT and reverse: 5′-CGGGGCAGTGCAAATGTTAAAA; *miR-145*, forward: 5′-GTCGTATCCAGTGCGCAGUUUCCAGGAAUCCCUUAATTGCACTGGATACGACCGCATT and reverse: 5′-GUCCAGUUUUCCCAGGAAUCCC; *miR-1305*, forward: 5′- ACAGGCCGGGACAAGTGCAATA and reverse: 5′-GCTGTCAACGATACGCTACGTAACG; U6, forward: 5′-AACGCTTCACGAATTTGCGT and reverse: 5′-CTCGCTTCGGCAGCACA. The experiment was performed in triplicate.

### Quantitative real-time PCR to detect the mRNA expression level

Total RNA was extracted with the TRIzol reagent (Invitrogen). The reverse transcription was performed with the PrimeScript First Strand cDNA Synthesis Kit according to the manufacturer’s instructions. The quantitative PCR reaction was carried out with the ABI 7500 System with the previously established protocol. Glyceraldehyde-3-phosphate dehydrogenase (GAPDH) was used as the endogenous control. The primers used are listed as follows: Smad4, forward: 5′-TGACCACGCCGTCTTCGTGC and reverse: 5′-CTGCTGCTGCATCTGCCGGT; GAPDH, forward: 5′-AGACAGCCGCATCTTCTTGT and reverse: 5′-CTTGCCGTGGGTAGAGTCAT.

### Microarray analysis

MiRNA were isolated from SMSCs expressing DANCR or the control vector using the miRcute miRNA isolation kit (DP501, TIANGEN Biotech (Beijing) Co., Ltd.). The quality of miRNA was detected by the NanoDrop2000 (Thermo Fisher Scientific, Inc.). MiRNA microarray analysis was performed using the Agilent human miRNA 21.0 chip (design ID: 070156, Agilent Technologies, Inc.). The array images were obtained with the Feature Extraction software (version number: 10.7.1.1; Agilent Technologies, Inc.). GeneSpring GX software (version number: 12.5; Agilent Technologies, Inc.) was applied for normalization and analysis of the raw data. The cut-off value for the down- or up-regulation was set at 2.0.

### Western blot

SMSCs were harvested and washed with precooled PBS. Cells were lysed with the RIPA buffer (50 mM Tris/HCl, pH 8.0, 150 mM NaCl, 1% nonidet-P40, 1% sodium deoxycholate, and 0.1% SDS). The protein concentration was determined with the BCA kit (Thermo Scientific). Thirty micrograms of protein was loaded in each lane of the SDS/PAGE (10% gel) (Bio–Rad) and then transferred on to the PVDF membrane. The membrane was blocked with 5% BSA for 1 h at room temperature and then incubated with the indicated primary antibodies against Smad4 (Abcam) and β-actin (Santa Cruz). The membrane was then incubated with HRP–conjugated secondary antibodies for 1 h at room temperature. The band was visualized by putting ECL at the top and bottom.

### Statistical analysis

Data were represented as mean ± S.D. from three independent experiments. Statistical analysis was performed using Student’s *t*test, ANOVA test using SPSS17.0 (SPSS. Inc., Chicago, IL, U.S.A.). *P*<0.05 was considered as significant difference.

## Results

### DANCR promotes SMSCs’ viability and chondrogenic differentiation

**Figure 1:**
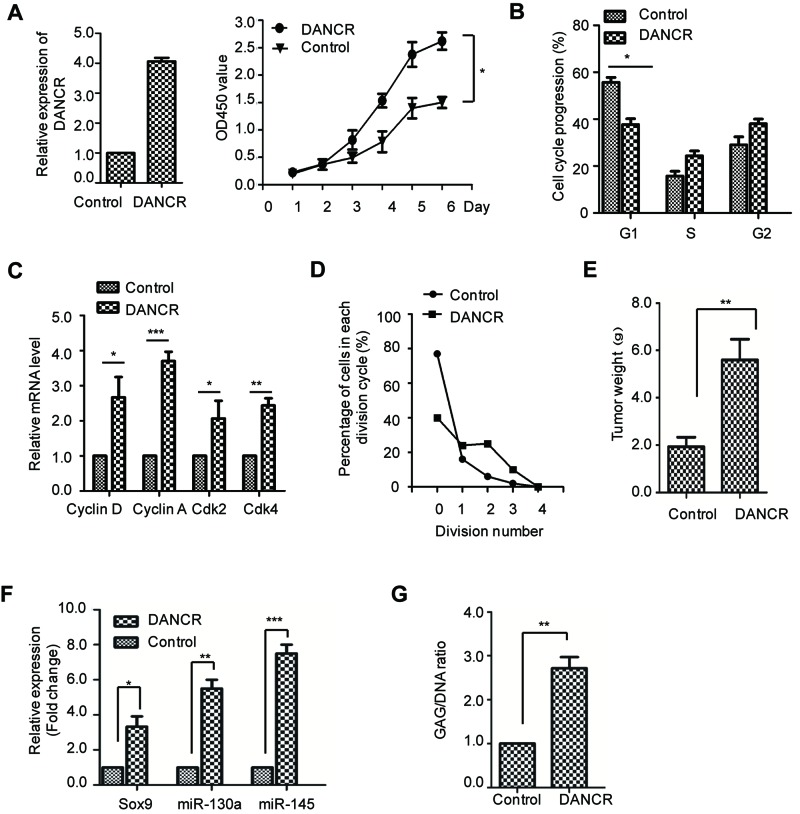
DANCR promotes the cell proliferation and chondrogensis in SMSCs (**A**) The cell proliferation rate of SMSCs harboring control vector or DANCR was detected with the CCK-8 assay with the absorbance at 450 nm. Results were presented as mean ± S.D. The significance *P<0.05 was determined by ANOVA test. The overexpression level of DANCR was detected by real-time quantitative RT-PCR (RT-qPCR) and shown as the left panel. (**B**) The cell cycle distribution of SMSCs with overexpressed DANCR or control vector was determined by FACS analysis. *P<0.05, Student’s *t* test. (**C**) The mRNA levels of the cell cycle regulators were detected by RT-qPCR. (**D**) The cell division of SMSCs with overexpressed DANCR or control vector was monitored. (**E**) SMSCs with overexpressed DANCR or control vector were injected into the nude mice. Mice were killed and the tumor was weighed; **P<0.01, Student’s *t* test. (**F**) The relative expression level of Sox9, *miR-130a*, and *miR-145* in SMSCs expressing control or DANCR were determined with RT-PCR analysis. Data were shown as mean ± S.D.; *P<0.05, **P<0.01, ***P<0.001, Student’s *t* test. (**G**) Biochemical analysis of the assessment of chondrogenesis index in SMSCs expressing control vector or DANCR. The glycosaminoglycan (GAG) content was normalized to the total DNA content; **P<0.01, Student’s *t* test.

To detect the effect of lncRNA DANCR on SMSCs, cell viability analysis was performed with SMSCs harboring DANCR or the control vector. As shown in Figure 1A, overexpression of lncRNA DANCR significantly increases the cell viability of SMSCs. As cell proliferation is tightly associated with cell cycle progression, the cell cycle distribution of SMSCs harboring control vector or DANCR was monitored by FACS analysis. The result showed that overexpression of DANCR resulted in decreased cell cycle distribution in G_1_-phase (Figure 1B), which suggested that highly expressed DANCR promoted the cell cycle progression. Consistently, the expression level of cell cycle regulators including Cyclin D, Cdk2, Cdk4, and Cyclin A were significantly increased in SMSCs with overexpressed DANCR (Figure 1C). Additionally, the cell division assay also demonstrated that highly expressed DANCR resulting in more cells entering division (Figure 1D). Collectively, these data demonstrated that DANCR promoted the cell proliferation of SMSCs. To confirm this observation, the promotion of DANCR on SMSC cell proliferation was also assessed by the *in vivo* nude mice tumorigenesis assay. SMSC stably expressing DANCR or control vector was subcutaneously injected into the nude mice, and the tumor formation was monitored. The result showed that SMSCs harboring DANCR generated tumors with increased tumor weight compared with that of the control cells (Figure 1E). These results indicated that overexpression of DANCR promotes the cell proliferation of SMSC both *in vitro* and *in vivo*.

To further evaluate the effect of DANCR on the chondrogenic differentiation, the pellets of SMSCs expressing DANCR or control vector were cultured in DMEM medium containing the components essential for the chondrogenesis. The chondrogenic differentiation was determined by detecting the expression of chondrogenic-specific markers including Sox9, *miR-130a*, and *miR-145*. The result showed that SMSCs with highly expressed DANCR exhibited significantly increased abundance of Sox9, *miR-130a*, and *miR-145* (Figure 1F). To further support this conclusion, the accumulation of GAG (glycosaminoglycan) was measured in DANCR-overexpressed SMSCs. As indicated in Figure 1G, the GAG/DNA ratio was obviously increased in SMSCs expressing DANCR in comparison with that of the control cells. Collectively, the above data suggested that lncRNA DANCR promotes the cell proliferation and chondrogenic differentiation of SMSCs.

### DANCR down-regulates *miR-1305*

**Figure 2:**
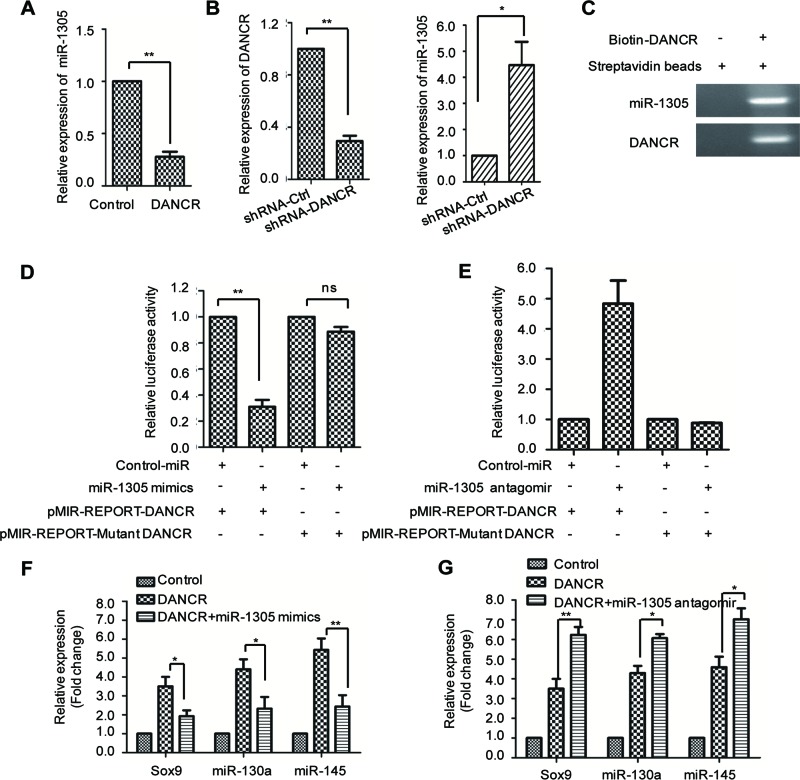
DANCR negatively regulates the expression of *miR-1305* (**A**) SMSCs were transfected with the expression plasmid of DANCR or the control vector. The relative expression abundance of *miR-1305* was detected with RT-PCR assay. **P<0.01, Student’s *t* test. (**B**) SMSCs were transfected with shRNA-DANCR or shRNA-control. The knockdown efficiency of DANCR (left panel) and the expression level of *miR-1305* (right panel) were determined. *P<0.05, Student’s *t* test. (**C**) Biotin-labeled DANCR were transfected into the SMSCs. Streptavidin beads were added and incubated with the cell lysis. The binding between DANCR and *miR-1305* was detected by PCR. (**D**,**E**) SMSCs were cotransfected with DANCR reporter vector in the presence of *miR-1305* mimics (D) or antagomir (E). Significantly decreased luciferase activity was observed with the transfection of *miR-1305* mimics (D). Depletion of *miR-1305* with *miR-1305* antagomir increased the luciferase activity (E); **P<0.01, Student’s *t* test. NS, no significant. (**F**,**G**) The relative expression levels of Sox9, *miR-130a*, and *miR-145* of SMSCs expressing the indicated vector were determined.

To explore whether miRNAs were involved in DANCR-facilitated SMSC growth and chondrogenesis, miRNA microassay analysis was carried out to search for the candidate miRNAs that were regulated by SMSC overexpression. A total of 500 miRNAs were screened using an Agilent human miRNA bioarray (design ID: 070156). The raw data were normalized with the quantile algorithm. Differentially expressed miRNAs were identified by fold change (FC) and the *P*-values were calculated by the Student’s *t* test. The cutoff for up- and down-regulation of genes was set as FC ≥2 and *P*<0.05. The data analysis showed that a total of 107 aberrantly expressed miRNAs in SMSCs with overexpressed DANCR, which includes 63 miRNAs that were up-regulated and 44 miRNAs that were down-regulated. Of the dysregulated miRNAs, *miR-1305* was the most down-regulated miRNA in SMSCs harboring DANCR compared with the control. To confirm this observation, the expression abundance of *miR-1305* with highly expressed DANCR was detected by real-time quantitative RT-PCR (RT-qPCR) and the result showed a significant decreased level of *miR-1305* in DANCR-overexpressed SMSCs (Figure 2A). Consistently, down-regulation of DANCR increased the expression level of *miR-1305* (Figure 2B). This observation suggested that *miR-1305* might serve as a downstream effector in DANCR-enhanced cell proliferation and chondrogenesis.

To further illustrate the regulatory relationship between DANCR and *miR-1305*, we detected the binding between DANCR and *miR-1305*. DANCR–conjugated with biotin was transfected into SMSCs, and then RNA immunoprecipitation (RIP) assay was performed. The result showed that *miR-1305* was detected in the immumocomplex enriched by DANCR, which suggested the interaction between DANCR and *miR-1305* (Figure 2C). To support this conclusion, luciferase activity was performed by constructing DANCR into the pMIR-REPORT vector. SMSCs were cotransfected with pMIR-REPORT-DANCR and *miR-1305* mimics or the control vector. The result showed that the luciferase activity was significantly decreased in the presence of *miR-1305*, while transfection of the mutant pMIR-REPORT-DANCR with *miR-1305* mimics had no obvious effect on the luciferase activity (Figure 2D). Consistent with this result, SMSCs were transfected with the *miR-1305* antagomir to inhibit the endogenous expression of *miR-1305*. Increased luciferase activity was observed with the suppression of *miR-1305* (Figure 2E). These results demonstrated the binding between *miR-1305* and DANCR.

As DANCR negatively regulates *miR-1305*, we hypothesized that whether highly expressed *miR-1305* could reverse the promoting effect of DANCR on chondrogensis. To this end, SMSCs stably expressing DANCR were transfected with *miR-1305* mimics and the chondrogenic differentiation was monitored. As shown in Figure 2F, overexpression of *miR-1305* abolished the up-regulated expression of Sox9, *miR-130a*, as well as *miR-145*, which indicated the inhibition of DANCR-facilitated chondrogenesis. Consistently, cotransfection of DANCR with miR-1305 antagomir further enhanced the effect of DANCR in chondrogenic differentiation (Figure 2G). These results demonstrated that up-regulation of *miR-1305* antagonized the effect of DANCR in chondrogenesis.

### Smad4 is the target of *miR-1305*

**Figure 3:**
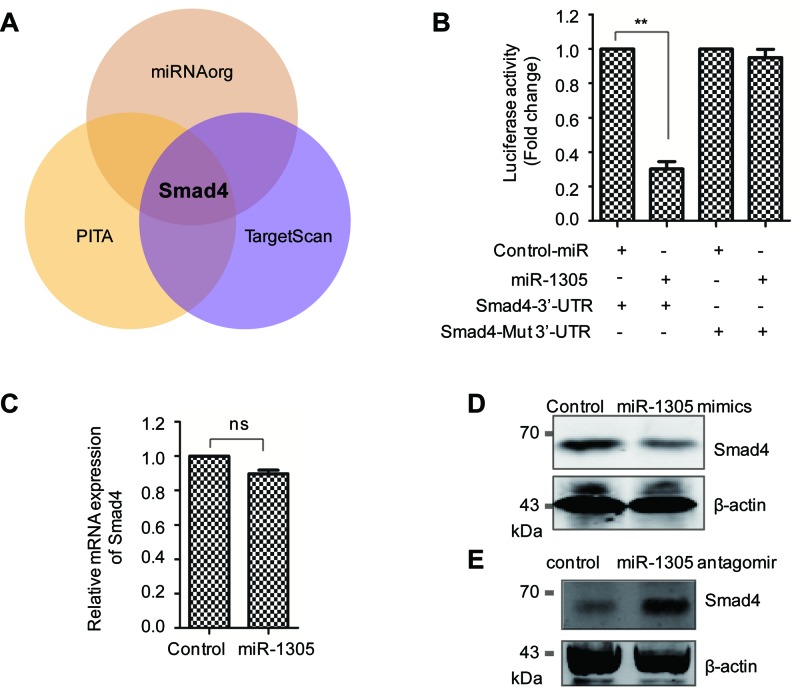
Smad4 is a target of *miR-1305* (**A**) The candidate downstream targets of *miR-1305* were predicted with the microRNAorg, PITA, and TargetScan databases. The Venn diagram showed that Smad4 is found as the intersection of the three databases. (**B**) SMSCs were transfected with the indicated vectors and the luciferase activity was monitored. (**C**) The mRNA expression level of Smad4 was detected by RT-PCR with SMSCs harboring control vector or *miR-1305* mimics; NS, no significant. (**D**,**E**) SMSCs were transfected with *miR-1305* mimics or *miR-1305* antagomir, and then the endogenous protein level of Smad4 was detected with anti-Smad4 antibody by Western blot.

It is well documented that the physiological function of miRNA is to induce the degradation or inhibit the translation of target mRNA [25]. To search for the downstream targets of miR-1305, TargetScan (www.targetscan.org), microRNAorg (http://www.microrna.org) and PITA algorithm (http://genie.weizmann.ac.il) databases were used to predict the candidate targets of *miR-1305*. Amongst the three databases, Smad4 was identified as the possible target gene with the ‘seed sequence’ in the 3′-UTR of Smad4 (Figure 3A).

To validate this, luciferase reporter assay was performed with SMSCs cotransfected with miR-1305 and either wild-type or mutant 3′-UTR of Smad4. As shown in Figure 3B, wild-type Smad4 showed decreased luciferase activity in the presence of *miR-1305*, whereas the relative luciferase activity in the mutant 3′-UTR of Smad4 was comparable with that of the scramble control cells. This result suggested that Smad4 is a target of *miR-1305*. To further confirm this conclusion, we investigated the protein and mRNA level of Smad4 with SMSCs expressing *miR-1305*. The result showed that the mRNA level of Smad4 was not significantly changed, while the protein abundance of Smad4 was down-regulated in SMSCs with highly expressed *miR-1305* (Figure 3C,D). In addition, the endogenous expression of *miR-1305* was depleted by transfecting *miR-1305* antagomir, and the protein level of Smad4 was detected by Western blot. As shown in Figure 3E, depletion of *miR-1305* increased the protein level of Smad4. These results indicated the negative regulatory relationship between *miR-1305* and Smad4.

### Smad4 is required for the function of DANCR in the cell proliferation and chondrogenic differentiation of SMSCs

**Figure 4:**
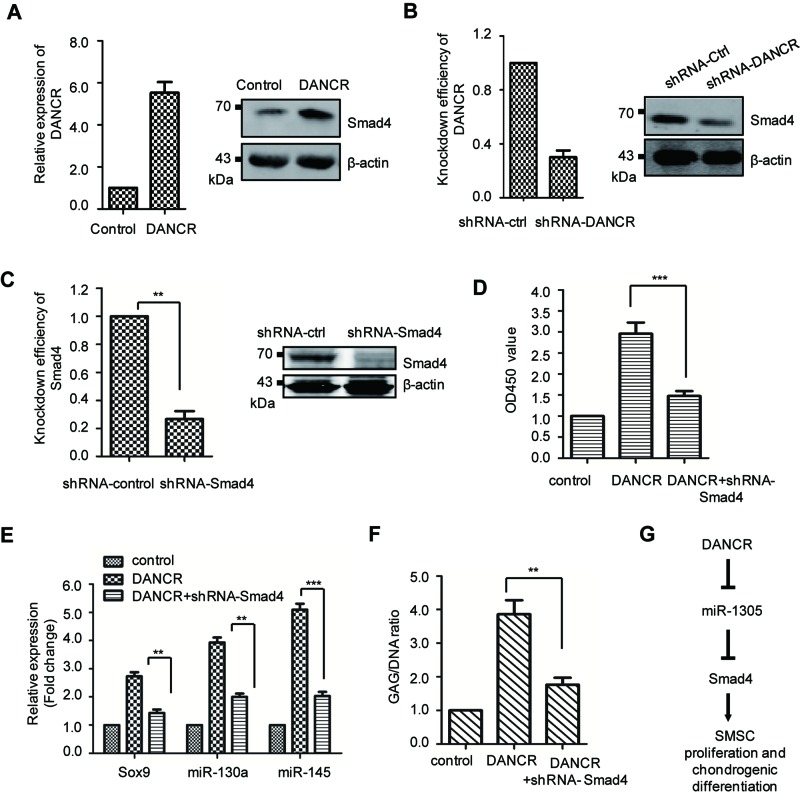
Smad4 is required for the functioning of DANCR in promoting cell proliferation and chondrogensis in SMSCs (**A**) SMSCs were transfected with control vector or DANCR. The level of DANCR was quantitated (left panel) and the protein level of Smad4 was detected with anti-Smad4 antibody (right panel). (**B**) The endogenous expression of DANCR was depleted with shRNA-DANCR (left panel) and the protein abundance of Smad4 (right) was determined. (**C**) The knockdown efficiency of Smad4 was detected at mRNA and protein level, respectively. (**D**) SMSCs expressing the indicated vector were cultured for 6 days and the cell viability was monitored at 450 nm; ***P<0.001, Student’s *t* test. (**E**) Relative expression level of the Sox9, *miR-130a*, and *miR-140* of SMSCs harboring the indicated expressing vector was detected. Data were represented as mean ± S.D. (**F**) Biochemical analysis of the assessment of chondrogenesis index in indicated cells. Total DNA content was used as the normalization; **P<0.01, Student’s *t* test. (**G**) The diagram to summarize the relationship amongst DANCR, *miR-1305*, and Smad4. Overexpression of DANCR down-regulates *miR-1305*. And *miR-1305* targetted and negatively regulated the expression of Smad4 in SMSCs. Collectively, DANCR regulated *miR-1305*-Smad4 axis in the cell proliferation and chondrogenic differentiation of SMSCs.

Our results showed that DANCR negative regulates the expression of *miR-1305* and Smad4 is a major target of *miR-1305*. To explore the effect of DANCR on Smad4, SMSCs were transfected with DANCR and the protein abundance of Smad4 was detected by Western blot. As shown in Figure 4A, overexpression of DANCR increased the expression level of Smad4. Consistently, down-regulation of DANCR reduced the abundance of Smad4 (Figure 4B). To further illustrate whether Smad4 is required for the functioning of DANCR, SMSCs were transfected with DANCR and the endogenous expression of Smad4 was depleted. The knockdown efficiency of Smad4 was shown in Figure 4C. The result showed that overexpression of DANCR enhanced the cell proliferation of SMSCs, while depletion of Smad4 abolished this effect of DANCR (Figure 4D). In addition, the expression of chondrogenic specific markers Sox9, *miR-130a*, and *miR-145* were also detected with the down-regulation of Smad4. As shown in Figure 4E, depletion of Smad4 decreased the expression level of Sox9, *miR-130a*, and *miR-145* that was induced by DANCR. Consistently, the accumulation of GAG was abolished in Smad4-depleted cells (Figure 4F). These data suggested that Smad4 is essential for the function of DANCR in the cell proliferation and chondrogenic differentiation of SMSCs.

## Discussion

Cartilage lesions caused by joint injuries do not heal well in adult as the poor regenerative capacity of cartilage. SMSCs have shown promising efficiency for cell-based regeneration of damaged articular cartilage [14]. Our previous data demonstrated that chondrogenic differentiation of SMSCs was promoted by lncRNA DANCR [12,13]. In the present study, we characterized the underlying molecular mechanism that overexpression of DANCR down-regulates *miR-1305*. Notably, overexpression of *miR-1305* suppressed the promotion of DANCR in the cell proliferation and chondrogenic differentiation of SMSC, suggesting the negative regulatory loop between DANCR and *miR-1305*. Further mechanism study uncovered that *miR-1305* targetted and negatively regulated the expression of Smad4 in SMSCs. These data indicated the DANCR regulated *miR-1305*-Smad4 axis in chondrogenic differentiation (Figure 4G).

MiRNAs regulate target gene expression by inducing mRNA degradation or suppressing mRNA translation through base-pairing with the 3′-UTR of the mRNA [26]. Increasing evidence has illustrated the essential roles of miRNA in the differentiation and development of bone and cartilage [29,31–33]. Up-regulated expressions of *miR-410, miR-99a*, and *miR-140* in early chondrogenic differentiation have been identified [34–36]. With miRNA microarray analysis, we found that the expression level of *miR-1305* was significantly decreased in DANCR-overexpressed SMSCs. Recent study demonstrated that down-regulation of miR-1305 facilitated the maintenance of pluripotency and increased the cell survival of human induced pluripotent stem cells (hiPSCs) [37]. These results point to an important role for *miR-1305* as a novel regulator of cell differentiation and proliferation.

Chondrogenic differentiation is potentially induced by TGF-β [38–40], a multifunctional cytokine that is involved in prenatal and postnatal development, maintenance of normal organ structure, and would healing. Smad4 is involved in TGF-β signaling via Smad2/3. The prochondrogenic effect of Smad2/3 on human MSCs has been well documented [41], while the function of Smad4 during chondrogenesis needs further investigation. In the present study, we found that *miR-1305* interacts with the 3′-UTR of Smad4; overexpression of *miR-1305* led to a significant reduction in Smad4 protein *expression*. These results identified Smad4 as a downstream target of *miR-1305* in chondrogenic differentiation of SMSCs. Recent study showed that knockdown of Smad4 in fetal BMSCs completely blocked chondrogenesis [42]; and mesenchyme-specific deletion Smad4 leads to an absence of the limb skeleton as a result of impaired chondrogenesis [43]. These studies demonstrated the important role of Smad4 in chondrogenic differentiation.

In conclusion, the present study demonstrated that miR-1305 was significantly decreased in SMSCs with the overexpression of DANCR. Down-regulation of *miR-1305* enhanced the chondrogensis of SMSCs through targetting Smad4. Consistently, highly expressed *miR-1305* suppressed the promotion of DANCR in cell proliferation and chondrogenic differentiation of SMSCs. These findings provided a novel mechanism of DANCR in chondrogenic differentiation and indicated that the control of *miR-1305*-Smad4 axis should be considered in the pathophysiology of cartilage regeneration.

## Abbreviations

CCK-8: cell counting kit-8
CFSE: carboxyfluorescein diacetate succinimidyl ester
FC: fold change
GAG: glycosaminoglycan
GAPDH: glyceraldehyde-3-phosphate dehydrogenase
lncRNA: long noncoding RNA
MSC: mesenchymal stem cell
RT-qPCR: real-time quantitative RT-PCR
SMSC: synovium-derived mesenchymal stem cell
Sox9: SRY-box 9
Cdk: cyclin-dependent kinase
STAT3: signal transducer and activator of transcription 3
